# Heat shock cognate protein 70 contributes to *Brucella *invasion into trophoblast giant cells that cause infectious abortion

**DOI:** 10.1186/1471-2180-8-212

**Published:** 2008-12-05

**Authors:** Kenta Watanabe, Masato Tachibana, Satoshi Tanaka, Hidefumi Furuoka, Motohiro Horiuchi, Hiroshi Suzuki, Masahisa Watarai

**Affiliations:** 1 Department of Veterinary Public Health, Faculty of Agriculture, Yamaguchi University, Yamaguchi, Japan; 2 Research Center for Protozoan Diseases, Obihiro University of Agriculture and Veterinary Medicine, Obihiro, Japan; 3 Department of Animal Resource Science and Veterinary Medical Science, the University of Tokyo, Tokyo, Japan; 4 Department of Veterinary Medical Science, Obihiro University of Agriculture and Veterinary Medicine, Obihiro, Japan; 5 Graduate School of Veterinary Medicine, Hokkaido University, Sapporo, Japan; 6 Department of Development and Medical Technology, the University of Tokyo, Tokyo, Japan

## Abstract

**Background:**

The cell tropism of *Brucella abortus*, a causative agent of brucellosis and facultative intracellular pathogen, in the placenta is thought to be a key event of infectious abortion, although the molecular mechanism for this is largely unknown. There is a higher degree of bacterial colonization in the placenta than in other organs and many bacteria are detected in trophoblast giant (TG) cells in the placenta. In the present study, we investigated mechanism of *B. abortus *invasion into TG cells.

**Results:**

We observed internalization and intracellular growth of *B. abortus *in cultured TG cells. A monoclonal antibody that inhibits bacterial internalization was isolated and this reacted with heat shock cognate protein 70 (Hsc70). Depletion and over expression of Hsc70 in TG cells inhibited and promoted bacterial internalization, respectively. IFN-γ receptor was expressed in TG cells and IFN-γ treatment enhanced the uptake of bacteria by TG cells. Administering the anti-Hsc70 antibody to pregnant mice served to prevent infectious abortion.

**Conclusion:**

*B. abortus *infection of TG cells in placenta is mediated by Hsc70, and that such infection leads to infectious abortion.

## Background

Brucellosis is a widespread and economically important infectious disease of animals and humans caused by members of the genus *Brucella*. *Brucella *spp. are small gram-negative, facultative intracellular pathogens that cause abortion, retained placenta and infertility in numerous domestic and wild mammals, and a disease known as undulant fever in humans [1-3]. Transmission of *Brucella *spp. from infected animals to humans may be either direct or indirect. Direct transmission involves the respiratory, conjunctival and cutaneous routes, and is more important in people in close contact with infected animals. Indirect transmission is through the consumption of contaminated dairy products [3]. *Brucella *spp. occasionally causes spontaneous abortion in pregnant women [4].

There have been several histological studies on the placentas of *Brucella *infected animals [5]. Further, it has been found that *Brucella *internalizes into the caprine erythrophagocytic trophoblastic epithelial cells from the maternal circulation [6] and that the internalized bacteria replicate within the rough endoplasmic reticulum, resulting in secondary infection of adjacent trophoblastic epithelial cells [6,7]. Researches have also shown that after necrosis of infected trophoblasts, large numbers of brucellae are released, and proximity of the fetal capillaries in the ulcerated placenta to the lumenal bacteria has been proposed as the source of the fetal bacteremia and further placental infection [6,8]. However, the molecular mechanism of abortion induced by *Brucella *spp. remains unknown.

The mouse model, particularly that using the unpregnant mouse, has been used extensively to study some aspects of the pathogenesis of brucellosis [2]. While brucellosis is known to primarily affect the reproductive tract in the natural host, little is known regarding the cellular and molecular mechanisms of *Brucella *infection in the pregnant mouse [9]. Although the structure of bovine placenta is completely different from mouse placenta, the infectious abortion model using the pregnant mouse is a powerful tool for investigating the mechanisms of *Brucella *pathogenesis. In our previous study, we demonstrated that *B. abortus *causes abortion in pregnant mice by inoculating bacteria on day 4.5 of gestation [10]. We found that there was a higher degree of bacterial colonization in the placenta than in other organs, that there were many bacteria in trophoblast giant (TG) cells in the placenta and that an intracellular replication-defective mutant did not induce abortion. These findings suggest that bacterial infection of TG cells plays a key role in abortion induced by *B. abortus *infection.

Pregnancy leads to a generalized suppression of the adaptive immune system, typified by significantly decreased cell-mediated immunity and reduced T helper cell (Th) 1 responsiveness [11-13]. This immunosuppressed state prevents maternal rejection of the fetus but has the unfortunate consequence of increasing maternal susceptibility to certain infectious agents [14,15]. Our previous study showed that a transient increase in interferon (IFN)-γ due to *Brucella *infection contributes to abortion in pregnant mice [10]. In addition to examining the balance of inflammatory and regulatory cytokines in bacteria infected pregnant mice, analysis of bacterial internalization into the TG cells, a specific host cells in placenta, will help to advance our knowledge regarding the control of *Brucella*-induced abortion.

In the present study, we investigated the internalization of *B. abortus *into TG cells and identified heat shock cognate protein 70 (Hsc70) as a candidate receptor against *Brucella *or bacterial uptake-associated molecule. We noted that IFN-γ enhances bacterial internalization into TG cells.

## Methods

### Bacterial strains

All *B. abortus *derivatives were from 544 (ATCC23448) smooth virulent *B. abortus *biovar 1 strains. GFP expressed 544 strain was used in this study [16,17]. *B. abortus *strains were maintained as frozen glycerol stocks and cultured on Brucella broth (Becton Dickinson) or Brucella broth containing 1.5% agar.

### Mice

Six to ten-week-old ICR female mice were individually mated to 6- to 10-week-old ICR male mice. The parent mice were obtained from CLEA Japan. Day 0.5 of gestation was the day the vaginal plug was observed. The normal gestational time for these mice is 19 days.

### Virulence in pregnant mice

Groups of five pregnant mice were infected intraperitoneally with approximately 10^4 ^colony forming unit (CFU) of brucellae in 0.1 ml saline on day 4.5 of gestation [10]. On day 18.5 of gestation, placenta and spleen were removed and homogenized in phosphate buffered saline (PBS). Tissue homogenates were serially diluted with PBS and plated on Brucella agar to count the number of CFU in each organ. Fetuses were determined to be alive if there was a heartbeat, and dead if there was no heartbeat. The animal experiments were permitted by Animal Research Committee of Obihiro University of Agriculture and Veterinary Medicine.

### Cell culture

Trophoblast stem (TS) cells were cultured in TS medium in the presence of FGF4, heparin and mouse embryonic fibroblast (MEFs)-conditioned medium as described previously [18]. The TS medium was prepared by adding 20% fetal bovine serum (FBS), 1 mM sodium pyruvate, 100 μM β-mercaptoethanol, and 2 mM L-glutamine to RPMI 1640. To induce differentiation to trophoblast giant (TG) cells, the cells were cultured in the only TS medium alone for 3 days at 37°C in CO_2 _incubator. The TG cells were seeded (1–2 × 10^5 ^per well) in 48 well tissue culture plates for all assays.

### Efficiency of bacterial internalization and replication within cultured cells

Bacterial infection and intracellular survival assays were performed according to a modified version of the method of Kim *et al *[19]. *B. abortus *strains were deposited onto TS or TG cells at a multiplicity of infection (MOI) of 100 which had been grown on 48-well microtiter plates containing TS medium but no antibiotics by centrifugation at 150 × g for 10 min at room temperature. To measure bacterial internalization efficiency after 30 min of incubation at 37°C, the cells were washed once with TS medium and then incubated with TS medium containing gentamicin (30 μg/ml) for 30 min. Next, cells were washed three times with PBS and lysed with cold distilled water. CFU values were determined by serial dilution on Brucella plates. To measure intracellular replication efficiency, infected cells were incubated at 37°C for 30 min, washed once with TS medium and then incubated with TS medium containing gentamicin (30 μg/ml) for 2, 24, 48 and 72 h. The cell washing, lysis and plating procedures were the same as for the bacterial internalization efficiency assay. Percentage protection was determined by dividing the number of bacteria surviving by the number in the infectious inoculum. The purified the R2–25 antibody or recombinant IFN-γ (Cedarlane Laboratories) was added to the TS medium at the indicated concentrations 2 or 12 h before infection.

### F-actin staining

GFP-expressing bacteria were deposited onto the cultured cells by centrifugation and the incubation was conducted at 37°C for 30 min. The infected cells were incubated with TS medium containing gentamicin (30 μg/ml) at 37°C for 30 min to kill extracellular bacteria and were then fixed in 4% paraformaldehyde for 30 min at room temperature. Next, samples were permeabilized in 0.2% Triton X-100, washed three times with PBS and incubated with Alexa Fluor 594-phalloidin (Molecular Probes) at 20 μg/ml for 30 min at 37°C. After three washes with PBS, samples were placed in mounting medium (90% glycerol containing 1 mg/ml phenylenediamene in PBS, pH 9.0) and visualized by fluorescence microscopy.

### Isolation of monoclonal antibodies

Hybridomas producing monoclonal antibodies that inhibit bacterial internalization into TG cells were obtained from fusions of BALB/c P3-X63-Ag8.653 (8-azaguanine-resistant and non-producer cell line) myeloma cells with spleen cells from Wister rats that had been immunized with TG cells. The screening of hybridoma supernatants for inhibiting antibodies was performed by adding antibodies to the TS medium in a bacterial internalization assay. Monoclonal antibodies obtained from hybridoma supernatants were purified using a protein G column (GE Healthcare Life Science) and the class and subclass of the purified monoclonal antibodies were determined using an Immunogloblin Typing Kit (WAKO Pure Chemical). The R2–25 monoclonal antibody used in this study was typed as IgG1.

### Subcellular fractionation of TS and TG cells

TS and TG cells (3 × 10^5^/ml) were seeded into each well of a 6-well plate. Protein isolation for the cytoskeleton, nuclear, membrane, and cytosol fraction was performed using a ProteoExtract Subcellular Proteome Extraction Kit as described by the manufacturer (Calbiochem).

### Immunoblotting

The cell lysates (500 μg/ml) and fractionated proteins (50 μg/ml) were separated on 10% polyacrylamide gels and transferred to a PVDF membrane, which was incubated for 1 h at room temperature with primary antibody (0.5 μg/ml) in 5% skim milk. It was then washed three times in Tris buffered saline (TBS) with 0.02% Tween 20, incubated for 30 min with a horseradish peroxidase (HRP)-conjugated secondary antibody at 0.01 μg/ml and then washed again. Immunoreactions were visualized by ECL (GE Healthcare Life Science). Antibodies for β-actin, β-tublin and histone H1 were purchased from SIGMA or Abcam. Anti-IFN-γ receptor rabbit polyclonal antibody was purchased from Santa Cruz Biotechnology.

### Mass spectrometry analysis

Identification of proteins reacting with monoclonal antibodies that inhibited bacterial internalization into TG cells was conducted by means of nano LC-MS/MS analysis and a search of MASCOT database (APRO life Science Institute, Japan).

### RNA isolation and RT-PCR

The total RNA of TG cells was isolated using an RNA Purification Kit (Qiagen) and purified RNA samples were stored at -30°C until use. The RNA was quantified by absorption at 260 nm using a SmartSpec3000 spectrophotometer (Bio-Rad). RT-PCR was carried out using a Sperscript II Kit (Invitrogen). The primers used for mouse Hsc70 or β-actin amplification had the following sequence 5'-GCAGCTGGGCCTACACACAAG-3' and 5'-CCCTGTGGAACAAAGCTACAC-3', or 5'-CGTGACATTAAGGAGAAGCTGTGC-3' and 5'-CTCAGGAGGAGCAATGATCTTGAT.

### Expression and purification of recombinant proteins

Mouse Hsc70 cDNA (GenBank Accession No. BC066191) was amplified from RNA isolated from TG cells by means of RT-PCR with the pair of primers described above. The product was cloned into the pCR2.1-TOPO vector (Invitrogen) (pCR-Hsc70). To achieve expression of recombinant Hsc70 protein, amplified DNA encoding Hsc70 from pCR-Hsc70 in PCR was cloned into pCold TF vector (Takara Bio Inc.). The His-tagged Hsc70 was expressed in the *E. coli *strain DH5α, and its purification and cleavage of His-tagged by HRV 3C protease were performed as described by the manufacturer (Novagen). Bovine Hsc70 and the rat anti-Hsc70 monoclonal antibody (SPA-815) were obtained from Stressgen for use as control materials.

To achieve expression of Hsc70 in TG cells, amplified DNA encoding Hsc70 from pCR-Hsc70 in PCR was cloned into the pcDNA4/TO vector in the T-Rex System (Invitrogen). pcDNA4/TO-Hsc70 was transfected into TG cells using the FuGENE 6 Transfection Reagent (Roche) with a final concentration of 1.2 μg/ml.

### siRNA experiment

The siRNA duplexes used for silencing mouse Hsc70 (target sequence: AACAAGTAACATGGAATAATA), and β-actin (target sequence: CACTGACTTGAGACCAATAAA) and AllStars Negative Control siRNA were purchased from QIGEN. TG cells were transiently transfected using oligofectamine (Invitrogen) with or without a final concentration of 10 nM for siRNAs.

### Immunofluorescence microscopy

Samples grown on coverslips were washed twice with PBS, fixed with 4% paraformaldehyde in PBS for 30 min at room temperature, and permeabilized with or without 0.2% Triton X-100 in PBS for 20 min at room temperature. After blocking with 5% BSA in PBS, the cells were incubated with primary antibody (25 μg/ml) for 1 h at 37°C, and detection was conducted with TRITC-labeled goat anti-rat IgG (0.01 μg/ml) (Chemicon). Fluorescent images were taken using an Olympus BX51 microscope and a cooled CCD camera Olympus DP70.

### *In vivo *depletion of Hsc70

Hsc70 was neutralized in the mice by administering an anti-mouse Hsc70 monoclonal antibody (clone R2–25)*in vivo *using 100 or 200 μg of antibody in a volume of 0.3 or 0.6 ml intraperitoneally 24 h before infection. Control mice were given 100 μg of normal rat IgG in a volume of 0.1 ml according to the same injection schedule as used for the anti-Hsc70 monoclonal antibody treated groups. Bacterial infection was conducted as described previously. On day 18.5 of gestation, fetuses were removed from the mice and a judgment made as to whether they were pregnant or not. Fetuses were determined to be alive if there was a heartbeat, and dead if there was no heartbeat.

### Statistical analysis

All statistical analysis was conducted using the Student *t *test.

## Results

### *B. abortus *internalizes and replicates in trophoblast giant cells

**Figure 1:**
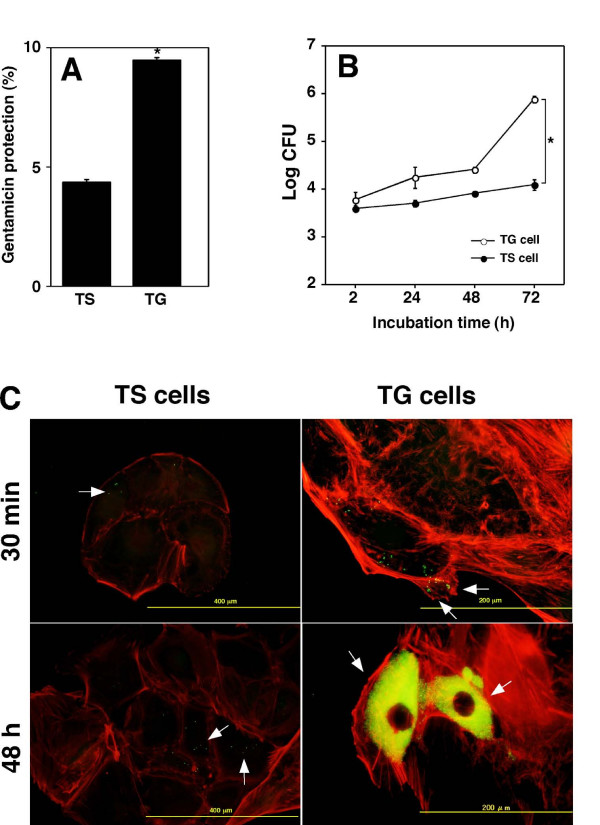
***B. abortus *infection in trophoblast giant cells**. (A) Bacterial internalization into trophoblast stem (TS) and giant (TG) cells. Data are the averages of triplicate samples from three identical experiments, and the error bars represent the standard deviations. Statistically significant difference between the bacterial internalization into TS and TG cells is indicated by asterisk (*, P < 0.01). (B) Intracellular replication of *B. abortus *in TS and TG cells. Datum points and error bars represent the mean of CFU of triplicate samples from a typical experiment (performed at least four times) and their standard deviations. Statistically significant difference between bacterial replication of *B. abortus *in TS and TG cells after 72 h of inoculation is indicated by asterisk (*, P < 0.01). (C) F-actin staining of bacteria infected cells. The figure shows GFP (bacteria) and Alexa Fluor 594 (actin filaments) channel merged images. Arrows indicate TS and TG cells containing bacteria.

We previously reported that there were many bacteria in trophoblast giant (TG) cells in the placenta by inoculation of pregnant mice with *B. abortus *[10]. To examine this bacterial infection into TG cells further, we used *in vitro *cell culture system of trophoblast stem (TS) cells and TG cells differentiated from TS cells. The *B. abortus *internalized into TG cells more efficiently than TS cells (Fig. 1A). We also investigated the intracellular replication of *B. abortus *in TS and TG cells. The bacteria replicated more efficiently in TG cells than TS cells (Fig. 1B).

Several intracellular pathogens attached to the host plasma membrane induce actin polymerization around the site of bacterial attachment and the process is essential for bacterial entry [20]. We therefore examined actin polymerization by means of fluorescence microscopy after 30 min and 48 h of incubation of TS and TG cells infected with *B. abortus*. It has been noted that differentiated TG cells dramatically rearrange their actin cytoskeleton into thick bundles of stress fibers [21]. There was no apparent actin polymerization around the site of the bacterial entry after 30 min incubation on TG cells or 48 h of incubation of infected TG cells (Fig. 1C).

### Isolation of monoclonal antibodies that inhibit bacterial internalization into TG cells

**Figure 2:**
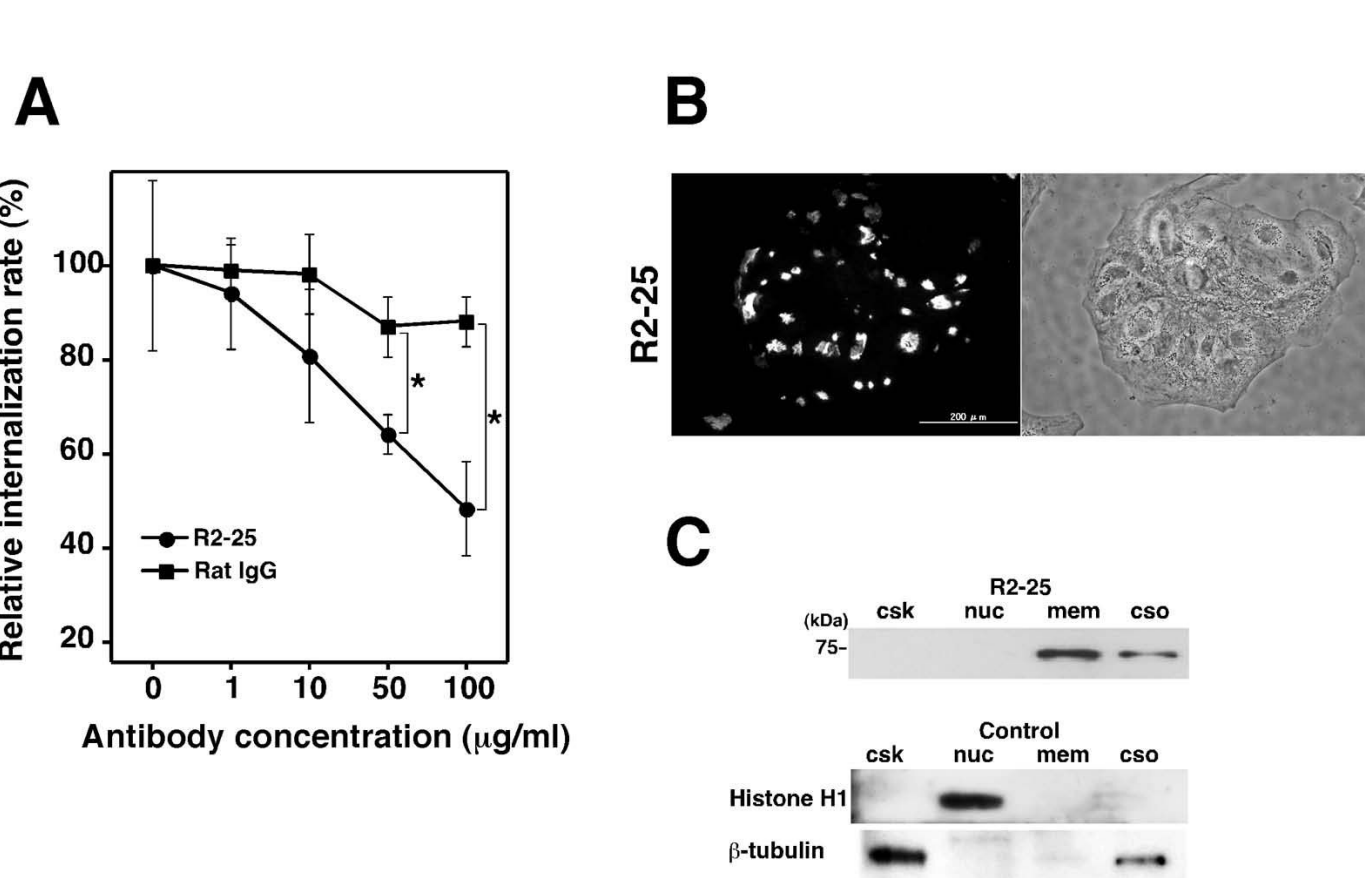
**Characterization of the monoclonal antibody that inhibits bacterial internalization into TG cells**. (A) Inhibition of bacterial internalization by the R2–25 antibody treatment. Data are the averages of triplicate samples from three identical experiments, and the error bars represent the standard deviations. Statistically significant differences between bacterial internalization into TG cells treated with the R2–25 antibody and those treated with rat IgG are indicated by asterisks (*, P < 0.01). (B) Distribution of protein reacting with monoclonal antibodies in TG cells. The left panels show fluorescence microscopy of the antibody stained TG cells and the right panels phase contrast microscopy of the corresponding microscopic fields. (C) Immunoblot analysis was performed on TG cell subcellular fractions with the monoclonal antibodies R2–25. Cells were fractionated to cytoskeleton (csk), nucleus (nuc), membrane (mem) and cytosol (cso). The anti-histone H1 and anti-β-tubulin antibody were used for fraction control for the nucleus and cytoskeleton.

To identify a receptor against *B. abortus *on TG cells, rats were immunized with TG cells, and monoclonal antibodies that inhibit bacterial internalization into TG cells were isolated. Seventy-five clones of bacterial internalization inhibiting antibodies (3.9%) were isolated from 1,920 hybridoma supernatants. From among the monoclonal antibodies, we selected R2–25, since it significantly inhibited internalization and showed clear reactions with protein in immunoblotting. The purified R2–25 antibody significantly inhibited bacterial internalization concentration dependently, but there was no inhibition with rat IgG (negative control) (Fig. 2A). The R2–25 antibody reacted with protein of around 70 kDa which was localized in membrane and cytosol, with the protein amount in the membrane fraction being especially large (Fig. 2C). On examining the distribution of protein reacting with R2–25 on the surface of TG cells by immunofluorescence microscopy, we observed an islet-like distribution on the surface of TG cells which was no permeabilized cells (Fig. 2B). It was difficult to detect the protein reacting with the R2–25 antibody on the surface of permeabilized cells.

### Antibody inhibiting bacterial internalization reacts with heat shock cognate protein 70

**Figure 3:**
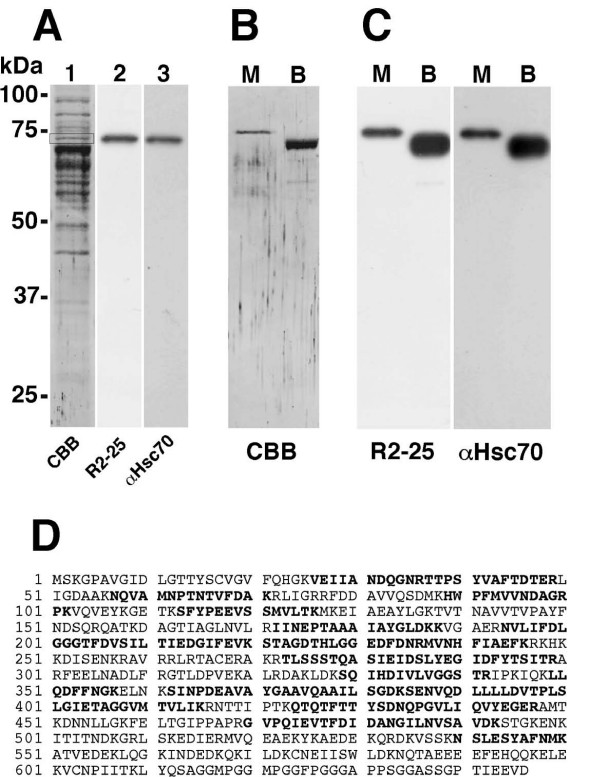
**Bacterial internalization inhibiting antibodies react with Hsc70**. (A) A membrane to which TG cell membrane fraction proteins had been transferred was stained with Coomassie brilliant blue (CBB) (lane 1), immunoblotted with the R2–25 antibody (lane 2) and the anti-Hsc70 antibody (clone SPA-815) (lane 3). (B) CBB staining of membrane to which mouse (M) or bovine (B) recombinant Hsc70 had been transferred. (C) Immunoblot analysis of mouse (M) or bovine (B) recombinant Hsc70 with R2–25 antibody and anti-Hsc70 antibody (clone SPA-815). (D) Amino acid sequences of Hsc70. The protein reacting with R2–25 was extracted from the PVDF membrane (square in lane 1 of panel A) and was used for LC-MS/MS analysis. The amino acids detected for Hsc70 in the analysis are shown in bold.

We performed mass spectrometry analysis to identify the protein reacting with the R2–25 antibody. Proteins of the membrane fraction were separated by SDS-PAGE and transferred to a PVDF membrane (Fig. 3A). The protein reacting with R2–25 was extracted from the PVDF membrane and the subjected to LC-MS/MS analysis. Through a search of the MASCOT database, the protein reacting with R2–25 was determined to be heat shock cognate protein 70 (Hsc70) (Fig. 3D). The anti-Hsc70 antibody also reacted with this protein (Fig. 3A). To confirm that the R2–25 antibody reacted with Hsc70, we tested its reactivity with mouse and bovine recombinant Hsc70. As expected, the R2–25 and anti-Hsc70 antibodies reacted with both types of recombinant Hsc70 (Fig. 3B and 3C). The mouse recombinant Hsc70 had a slightly greater molecular weight than the bovine recombinant Hsc70 because 25 amino acid residues had been added to the former after HRV 3C protease cleavage. These results indicate that the protein reacting with R2–25 was Hsc70.

### Hsc70 contributes to bacterial internalization into TG cells

**Figure 4:**
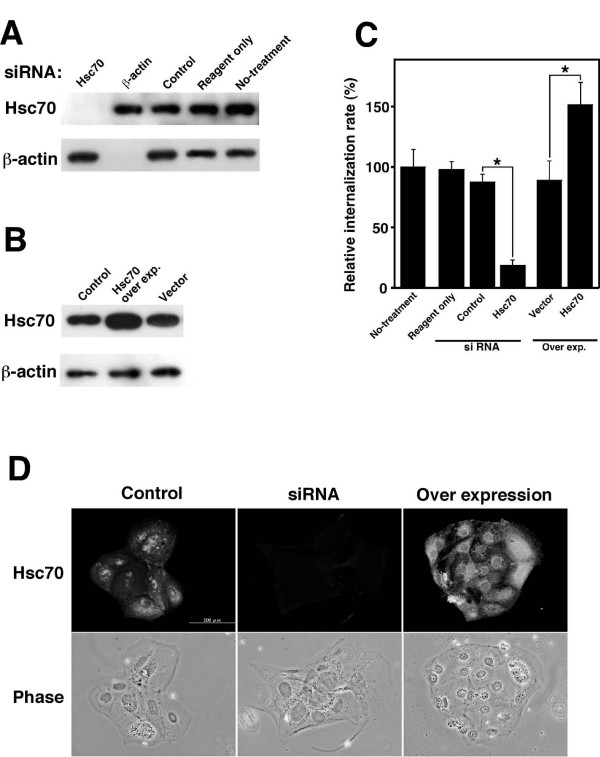
**Effect of depletion and over expression of Hsc70 in TG cells on bacterial internalization**. (A) Depletion of Hsc70. TG cells were treated for 48 h with siRNA targeting Hsc70 or without it (reagent only or no treatment), or β-actin or the control (QIAGEN AllStars Negative Control). Expression of the indicated proteins was monitored by immunoblotting. β-actin was used as an internal control. (B) Over expression of Hsc70. TG cells were transfected with or without (control) pcDNA4/TO-Hsc70 or vector only. (C) Bacterial internalization into Hsc70 depleted (siRNA) or over expressed (over exp.) TG cells was studied in a bacterial internalization assay. Lanes correspond to panels A and B. Data are the averages of triplicate samples from three identical experiments, and the error bars represent the standard deviations. Statistically significant differences between bacterial internalization into TG cells with (Hsc70) and without siRNA (control), and over expression and the control (vector) are indicated by asterisks (*, P < 0.01). (D) Distribution of Hsc70 in non-treated (control), Hsc70 depleted (siRNA), or over expressed (over expression) TG cells. Fluorescence microscopy of stained TG cells with the R2–25 antibody (upper panels) and phase contrast microscopy of the corresponding microscopic fields (lower panels) are shown.

To examine the effect of Hsc70 on bacterial internalization into TG cells further, we reduced the amount of endogenous Hsc70 by transfecting Hsc70-specific small interfering RNA (siRNA) duplexes into the TG cells. After 48 h of transfection with Hsc70-specific siRNA, the expression level of Hsc70 was no longer detectable, but was not affected by transfection with β-actin or the control siRNA (Fig. 4A and 4D). Thus, the internalization efficiency of *B. abortus *into TG cells was significantly reduced by transfection with Hsc70-specific siRNA (Fig. 4C). Next, excessive production of endogenous Hsc70 was induced by transfecting the Hsc70 expression vector into TG cells. After 48 h, expression levels of Hsc70 were significantly higher than the control levels (Fig. 4B and 4D). The internalization efficiency of *B. abortus *into TG cells in which Hsc70 was over-expressed was significantly higher than the control levels (Fig. 4C).

### IFN-γ enhances bacterial uptake by TG cells

**Figure 5:**
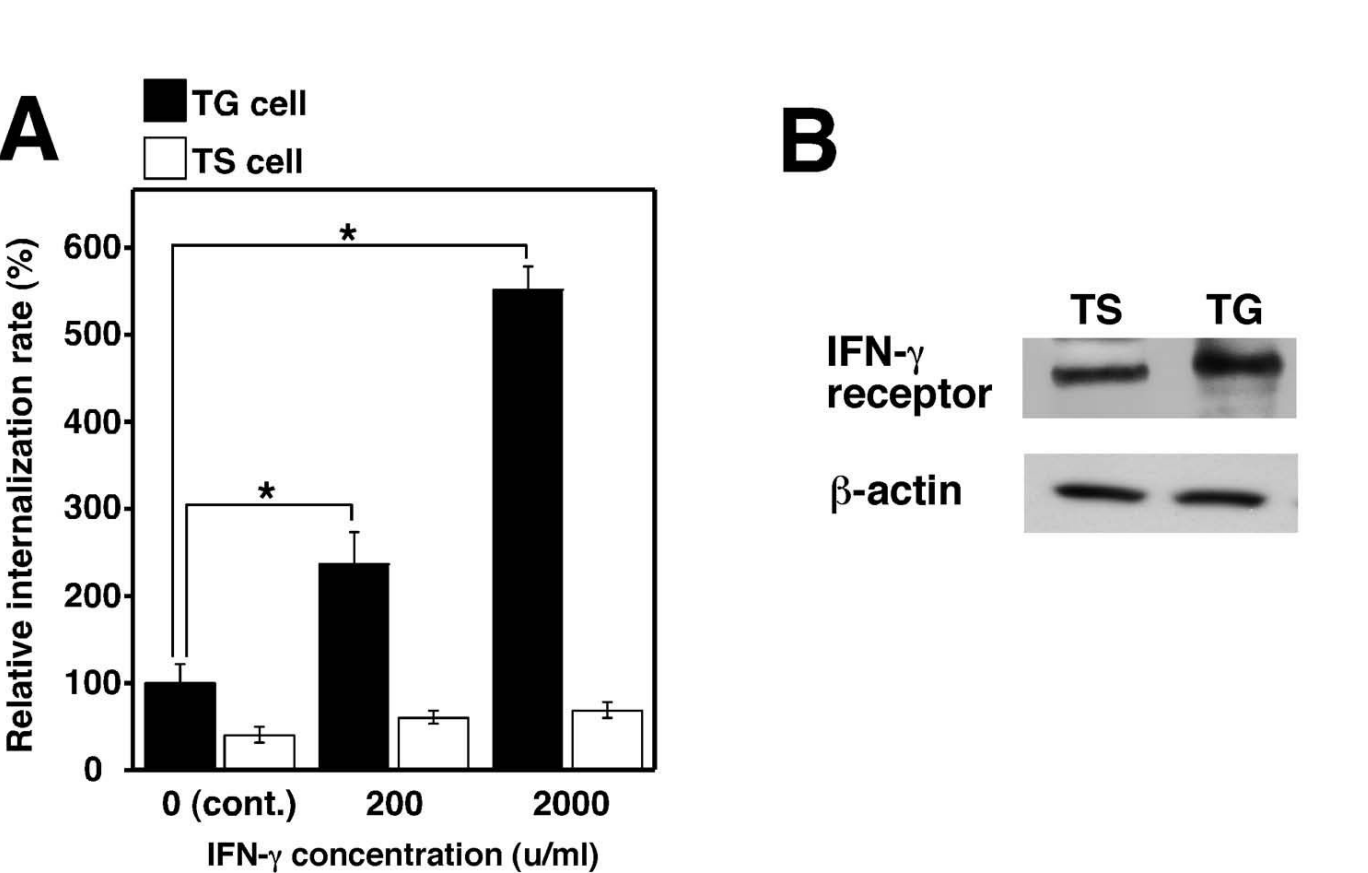
**IFN-γ promotes bacterial internalization into TG cells**. (A) Bacterial internalization into IFN-γ treated TS or TG cells. *B. abortus *was deposited onto TS and TG cells which were treated with or without (cont.) IFN-γ at the indicated concentrations. Data are the averages of triplicate samples from three identical experiments, and the error bars represent the standard deviations. Statistically significant differences between bacterial internalization in TG cells with and without IFN-γ treatment are indicated by asterisks (*, P < 0.01). (B) Expression of IFN-γ receptor in TS and TG cells. Immunoblot analysis was performed with anti-IFN-γ receptor and anti-β-actin rabbit polyclonal antibody.

Since a transient increase in IFN-γ brought about by *Brucella *infection promotes abortion in pregnant mice [10], we investigated the effect of IFN-γ treatment on bacterial internalization and Hsc70 expression in TG cells. IFN-γ treatment significantly increased the internalization efficiency of *B. abortus *into TG cells as their concentration, but had no effect in TS cells (Fig. 5A). To determine whether the enhancement of bacterial internalization by IFN-γ treatment was due to up-regulate Hsc70 expression or not, RNA was isolated from IFN-γ treated TG cells and subjected to RT-PCR. This showed that IFN-γ treatment did not affect Hsc70 expression (data not shown). IFN-γ receptor was expressed in TS and TG cells (Fig. 5B).

### Preventing abortion by inoculating pregnant mice with anti-Hsc70 antibody

**Figure 6:**
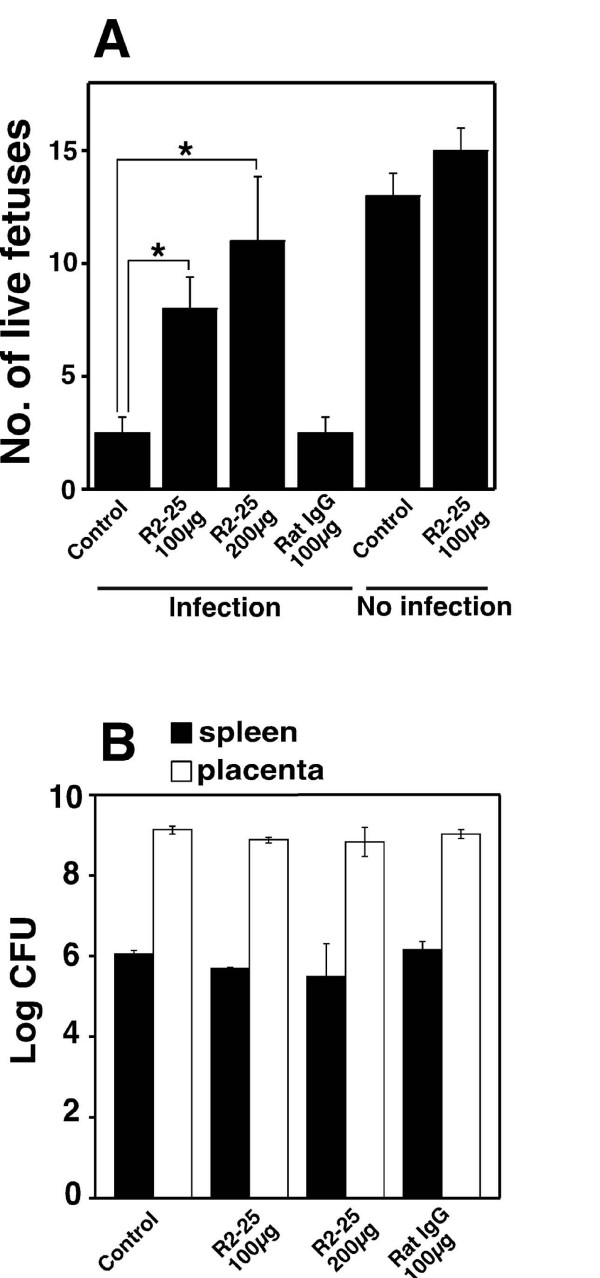
**Preventing abortion by inoculating with anti-Hsc70 antibody**. (A) Number of live fetuses. Hsc70 was neutralized in the mice by administering with or without (control) an anti-mouse Hsc70 monoclonal antibody (clone R2–25)*in vivo *using 100 or 200 μg of the antibody. The control mice were given 100 μg of normal rat IgG. Statistically significant differences between the untreated control and antibody treated mice are indicated by asterisks (*, P < 0.01). (B) Bacterial numbers in spleen and placenta. On day 18.5 of gestation, the placenta and spleen were removed and homogenized in PBS. Tissue homogenates were serially diluted with PBS and plated on Brucella agar in order to count the number of CFU in each organ.

To determine if abortion is prevented by neutralizing the Hsc70 expressed on TG cells in the mouse placenta, pregnant mice were inoculated with the R2–25 antibody 24 h before infection with *B. abortus*, which was done on day 4.5 of gestation. While there was no change in the number of abortions observed in the non-inoculated mice, there was a significant increase in number of live fetuses in the inoculated mice (Fig. 6A). Inoculation of uninfected pregnant mice with the R2–25 antibody did not affect on pregnancy (Fig. 6A). Upon examining bacterial numbers in the spleen and placenta of infected pregnant mice, it was found that bacterial numbers were similar in both mice inoculated with the R2–25 antibody and those not inoculated with it (Fig. 6B).

## Discussion

Previous mouse model studies have shown that *Brucella abortus *specifically replicates in trophoblast giant (TG) cells in the placenta [9,10]. TG cells are polyploid cells that play a crucial role in implantation, in remodeling of the embryonic cavity, and preventing maternal blood flow to the implantation site [22]. Since *B. abortus *internalizes into TG cells and replicates in them, cell functions are not exhibited completely, which leads to abortion since implantation and placental development are inhibited. Therefore, it is thought that bacterial infection of TG cells is a key event in inducing abortion. To analyze the molecular mechanisms of *B. abortus *infection of TG cells *in vitro*, we used trophoblast stem (TS) cells and TG cells differentiated from TS cells for the infection assay in this study. Although TG cell differentiation is fairly well understood at the morphological and molecular level [23], the role of immune responses in fighting against pathogens of TG cells is poorly understood and in this regard a model of host-pathogen interaction using TG cells would be useful for obtaining new information of the effect of TG cell functions on pregnancy.

Hsc70 has been reported to be present on the surface of several types of cells [24]. In this regard, though Hsc70 congregates on the surface of TG cells, it is present to a much lesser extent on the surface of TS cells (data not shown). This may be a reason that the internalization of *B. abortus *into TG cells was greater than that into TS cells. As Hsc70 and many other factors will be present on TG cells differentiated from TS cells, there is a possibility that other receptors or bacterial uptake-associated molecules may contribute to *B. abortus *infection of TG cells. Little is known about how Hsc70, a protein with no signal sequence for secretion, exits cells by mechanisms other than escape from cells undergoing necrotic lysis. In previous studies, Hsc70 has seen to be released from a late endsomal lysosomal location where it participates in protein degradation [25,26]. Further, the secretion of the Hsp70 family and its association with lipid rafts have also been observed in epithelial cells under normal conditions, and a lipid raft-based mechanism has been suggested for the membrane delivery and release of Hsp70 family [27]. Although receptors for the extracellular Hsp70 family have still not been fully defined, several cell surface receptors have been suggested, such as CD14, CD40, CD91 and scavenger receptor Lox-1 [28-31]. Since it has also been noted that class A scavenger receptor (SR-A) contributes to *B. abortus *infection in macrophages [32], SR-A may be receptors for Hsc70, and the mechanism for *B. abortus *internalization into TG cells may be the same pathway as that for Hsc70 uptake by TG cells. Hsc70 may have a function that is catching antigens and anti-Hsc70 would inhibit binding between Hsc70 and antigens. IFN-γ treatment enhanced bacterial internalization into TG cells and these observations agreed with results obtained in pregnant mice model [10], and thus expression of unidentified receptors against Hsc70 may be upregulated by IFN-γ treatment. IFN-γ should therefore promote internalization of *B. abortus *into TG cells *in vivo *and this would be one of ways in which infectious abortion is induced.

## Conclusion

The finding of this study that the anti-Hsc70 antibody prevents abortion caused by *B. abortus *infection is expected to be applied in the development of methods of preventing abortion. Since intracellular bacteria such as *Brucella *replicate in host cells, it is difficult to completely eliminate them from the host through treatment with antibiotics and develop effective vaccines against them. An alternative strategy in treating infection due to *Brucella *would be inhibition of bacterial internalization into TG cells and this could be an effective means of protecting against abortion due to brucellosis. Recently, Carvalho Neta *et al*. reported that *B. abortus *modulates innate immune response by bovine trophoblastic cells [33]. Although the structure of bovine placenta is completely different from mouse placenta, bovine and mouse trophoblastic cells may have similar function in the immune system. However, it is not known whether the mechanism of host-pathogen interaction observed in this study could be used to develop protective methods against other abortion-inducing pathogen infections, and thus further analysis of TG cell function in the immune system will be needed to clarify host defense mechanisms in the placenta and those contributing to the success of pregnancy.

## Authors' contributions

MW conceived the study. MW, HS and KW designed the experiments, interpreted the results and worked on the manuscript. KW and MT carried out most of the experimental work. ST, HF and MH participated in cell culture and pathological experiments. HS and MW participated in animal experiments. All authors read and approved the final manuscript.
